# Pivotal role of venous blood gas analysis in the detection of metabolic acidosis due to laxative abuse in an anorexia nervosa patient: A case report

**DOI:** 10.1002/pcn5.70055

**Published:** 2025-01-22

**Authors:** Satoshi Miwa, Takuto Ishida, Masafumi Mizuno

**Affiliations:** ^1^ Department of Psychiatry Tokyo Metropolitan Matsuzawa Hospital Tokyo Japan; ^2^ Department of Internal Medicine Tokyo Metropolitan Matsuzawa Hospital Tokyo Japan

**Keywords:** acidosis, anorexia nervosa, blood gas analysis, hypokalemia, laxative

## Abstract

**Background:**

Anorexia nervosa has the highest mortality rate of any psychiatric disorder, and purging behaviors can cause a fatal electrolyte and acid–base imbalance. Routine laboratory testing during inpatient care is essential because these patients often provide inaccurate information about their diet and purging behaviors. However, blood gas analysis for an acid–base evaluation is rarely performed in the psychiatric setting because psychiatrists are not accustomed to evaluating the results. This case highlights severe metabolic acidosis caused by excessive laxative use during inpatient care.

**Case Presentation:**

A 62‐year‐old female patient was admitted with suspected anorexia nervosa. She had stringently controlled her weight since her 20s, initiating laxative use in her 30s. In her 60s, she was referred to our hospital for suspected anorexia nervosa. On admission, her body mass index was 11.0 kg/m². Persistent complaints of severe constipation prompted an escalation in her laxative regimen. Abdominal radiographs demonstrated marked gas retention, corroborating her complaint. On hospital day 84, she abruptly lost consciousness with watery fecal incontinence. Her venous blood gas analysis demonstrated hyperchloremic metabolic acidosis caused by bicarbonate loss secondary to diarrhea. After regaining consciousness, she explained that watery stools were her typical bowel pattern. Discontinuation of laxatives ameliorated her condition.

**Conclusion:**

In this case, venous blood gas analysis was pivotal in detecting metabolic acidosis resulting from excessive laxative use. Physiological changes due to purging can be better evaluated by incorporating venous blood gas analysis into routine assessment. Further clinical studies are required to validate its utility in managing anorexia nervosa.

## Graphical abstract

The case report highlights the importance of routine venous blood gas analysis in psychiatric inpatient care for patients with anorexia nervosa, particularly in those with purging behaviors that risk dangerous acid–base imbalances. The case presented involves a 62‐year‐old woman with severe anorexia nervosa and chronic laxative use, which led to metabolic acidosis and loss of consciousness. It concludes that regular blood gas assessments may be beneficial in managing physiological complications in anorexia nervosa, advocating for further research on its integration into psychiatric care.

## BACKGROUND

Anorexia nervosa (AN) is common and has a prevalence of 0.3% among young women.^1^ This disease is characterized by starvation and malnutrition, and has the highest mortality rate of any psychiatric disease.^1^, ^2^, ^3^ AN patients with purging behaviors have an increased risk of suicide^4^ and the potential for various medical complications,^5^ which lead to a poorer prognosis than in AN patients without purging behaviors. Purging behaviors, such as self‐induced vomiting and excessive laxative use,^6^ lead to the development of marked defecation habits^7^ and abnormal laboratory findings, including electrolyte and acid–base imbalances.^8^ Metabolic alkalosis is the most common acid–base imbalance associated with these purging behaviors.^8^ On the other hand, reports of metabolic acidosis due to excessive laxative use are sparse, although metabolic acidosis is known to result from the loss of fluid and HCO_3_^−^ due to diarrhea. Furthermore, AN patients commonly make inaccurate statements about their weight, diet, and purging behaviors,^9^ thereby complicating the interpretation of the abnormal laboratory findings. Herein, we present a case of AN with purging behaviors in which escalation in laxatives regimen in response to self‐reported “severe constipation” resulted in severe metabolic acidosis with impaired consciousness.

## CASE PRESENTATION

A 62‐year‐old female patient was referred to our hospital for suspected AN following hospitalization for severe hypokalemia. She had begun a strict weight management program in her 20s. In her 30s, she began using laxatives to control her weight. As a result, her body mass index (BMI) decreased to 13 and her menstruation stopped. However, she did not seek medical attention for these problems because she denied being ill. She maintained the strict weight‐management regimen and continued taking laxatives for decades. In her 60s, she experienced a decline in health manifested by a mobility impairment which compelled her to crawl at home. She was later admitted to a hospital for severe hypokalemia (1.8 mEq/L) and suspected AN on the basis of her clinical history.

**Figure 1:**
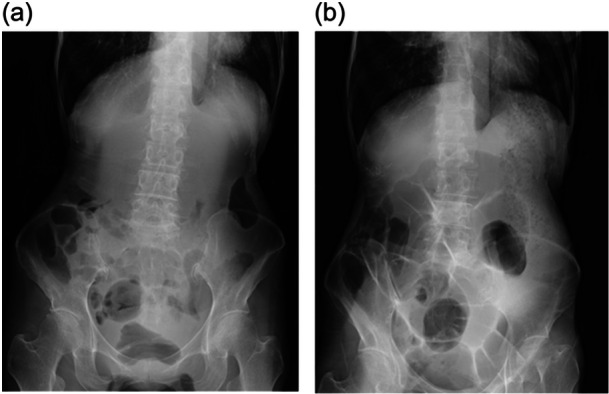
(a) Abdominal x‐ray on admission. (b) Abdominal x‐ray on hospital day 38.

On admission, her blood pressure was 79/51 mmHg and her other vital signs were unremarkable. Her BMI was 11.0 kg/m^2^. A physical examination revealed a 3 × 2‐cm decubitus ulcer on the sacral region, erythema on the internal and external condyles and heels, and a 2 × 2‐cm area of desquamation on the right elbow and right lower leg. The abdomen was flat and tympanic to percussion, with normal bowel sounds. A blood sample on admission revealed a corrected potassium level of 3.79 mEq/L with no other electrolyte imbalance (Table 1). Venous blood gas analysis demonstrated no abnormalities (Table 1). An abdominal radiograph found colonic gas retention (Figure 1a).

**Table 1:** Blood test and venous blood gas analysis (ambient air).

Item	On admission	Day 84
Albumin (g/dL)	2.22	3.56
Blood Urea Nitrogen (mg/dL)	9.8	29.4
Creatinine (mg/dL)	0.66	1.24
estimated Glomerular Filtration Rate (mL/min/1.73 m^−2^)	69.1	34.5
pH	7.416	7.082
PO_2_ (mmHg)	28.7	47.0
PCO_2_ (mmHg)	48.4	12.1
HCO_3_^−^ (mmHg)	30.4	3.5
Anion Gap	9.0	13.0
Glucose (mg/dL)	80.0	179.0
Lactate (mmol/L)	1.10	1.46
Na (mEq/L)	132.6	141.7
Cl (mEq/L)	97.0	129.0
K (mEq/L)	3.79	3.87

**Figure 2:**
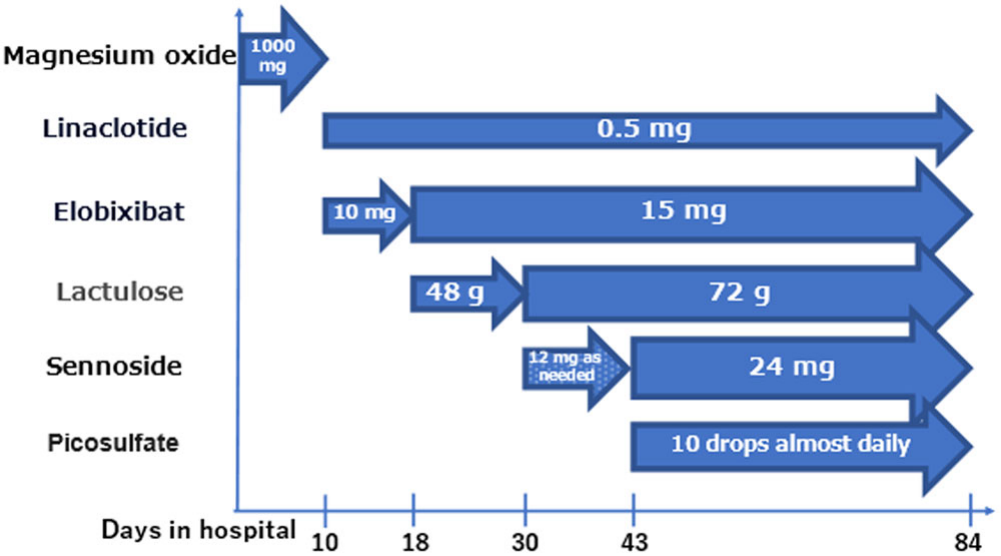
Progress of laxatives use.

Before admission, the patient had been managing her constipation with linaclotide 0.5 mg, sennoside 48 mg, and over‐the‐counter laxatives. After admission, treatment with linaclotide 0.5 mg and elobixibat 10 mg was begun. She complied with the hospital's diet during hospitalization. Her bowel movements were recorded daily in her medical charts, but she continued to complain that she had only a small amount of hard stools. On physical examination, the abdomen was flat, tympanic to percussion, and bowel sounds were normal on auscultation. An abdominal radiograph on hospital day 38 demonstrated residual fecal matter and colonic gas retention. These findings were consistent with her complaints and showed minimal improvement compared to the findings at admission (Figure 1b), therefore elobixibat 15 mg, lactulose 72 g, picosulfate solution 10 drops, and sennoside 24 mg were added in stages (Figure 2).

On hospital day 84, she experienced a sudden loss of consciousness. Her Glasgow Coma Scale score was E2 V1 M5 and her respiratory rate was 24/min. The other vital signs were unremarkable. At the same time, she presented with watery, fecal incontinence, which contradicted her previous complaints. Venous blood gas analysis revealed pH 7.082, anion gap 13.0, sodium 141.7 mEq/L, and chloride 129 mEq/L, indicating hyperchloremic metabolic acidosis (Table 1). A blood test demonstrated renal dysfunction and hyperchloremia, but hypokalemia was negative (Table 1). The hyperchloremic metabolic acidosis was primarily attributed to bicarbonate loss due to diarrhea, therefore the laxatives were discontinued and sodium bicarbonate was administered, resulting in an improvement in the metabolic acidosis. When she regained consciousness, she explained that watery stools were typical of her bowel movements. She stated, “I have always experienced loose stools; it has always been that way and is inherent to my constitution.” This statement indicated that she had considered laxative‐induced loose stools as a normal condition. The nurses documented the stool characteristics using the Bristol Stool Scale, relying exclusively on her self‐reported information without direct visual assessment. Consequently, we remained unaware that the stools were watery. Treatment with elobixibat 15 mg, lactulose 72 g, and picosulfate solution was resumed. As a result, the patient achieved Bristol Stool Form Scale 4–7 defecation approximately every 2 days and the venous blood gas analysis values normalized. She demonstrated a partial improvement in her awareness of her AN and expressed a willingness to cooperate in monitoring stool characteristics. However, she remarked “This is how I have always managed it. At my age, it would be challenging to change the habit.” Her attitude toward laxative abuse remained unchanged.

## DISCUSSION

The clinical course of this patient illustrates two important points in the treatment of AN. First, excessive use of laxatives can lead to fatal metabolic acidosis. Second, blood gas analysis should be performed to assess for an acid–base disturbance.

The most common acid–base disturbance in AN with purging behaviors is metabolic alkalosis.^8^ Metabolic alkalosis is commonly caused by the loss of Cl‐rich gastric acid due to self‐induced vomiting, which is the most common purging behavior in AN.^6^, ^8^ Purging behaviors can also cause hypokalemia, exacerbating the metabolic alkalosis.^10^, ^11^ Also, excessive laxative use in the presence of hypokalemia can lead to metabolic alkalosis.^12^ Hypokalemia disrupts the exchange system between HCO_3_^−^ secretion and Cl^−^ absorption in the intestinal tract and can induce the secretion of intestinal fluid characterized by a high Cl^−^ and low HCO_3_^−^ concentration.^12^ On the other hand, severe diarrhea commonly results in the loss of intestinal fluid, which contains a large amount of HCO_3_^−^. In turn, an increased compensatory renal reabsorption of Cl^−^ leads to hyperchloremic metabolic acidosis. However, cases of metabolic acidosis resulting from excessive laxative use are rare, possibly because AN with excessive laxative use often involves hypokalemia caused by purging behaviors. In the present case, the treatment for the hypokalemia may have contributed to the development of metabolic acidosis. Furthermore, previous studies reported that some cases of AN had been misdiagnosed as distal tubular acidosis owing to the similarity of the clinical presentation and the patients' concealment of excessive laxative use.^13^, ^14^ The present patient complained of persistent constipation and insisted on increasing the laxative dosage despite the watery diarrhea, which was actually her purging behavior. Clinicians should be aware that deliberate overdosing with laxatives can lead to fatal metabolic acidosis.

Venous blood gas analysis is useful for evaluating acid–base disturbances in AN patients, especially those with purging behaviors. Arterial blood gas analysis is the golden standard for assessing acid–base abnormalities, but routine arterial blood sampling is too invasive for the psychiatric setting. The pH and HCO_3_^−^ value on a venous blood gas analysis correlated with those on an arterial blood gas analysis, hence venous blood sampling is a useful substitute for the basic assessment of metabolic acid–base disturbance.^15^, ^16^

Evaluating the severity of purging behaviors is challenging because AN patients often make inaccurate statements.^9^ In objective assessment, the importance of abdominal examination and direct evaluation of stool characteristics by nurses is indisputable. However, AN patients, as observed in the present case, often consider laxative‐induced loose stools as a normal condition and strongly resist verification of stool characteristics if objective findings, such as abnormal blood tests results, are absent. Notably, our patient's reliance on laxatives remained unchanged even after experiencing fatal metabolic acidosis, as her defecation habit was deeply ingrained. In daily clinical practice, blood tests are often used to assess whether such diarrhea can be considered acceptable. However, a significant pitfall arises when electrolyte abnormalities are corrected through regular medication. In these cases, routine blood tests typically yield normal results, making it difficult to recognize severe acidosis without a venous blood gas analysis. In fact, despite the successful treatment of the hypokalemia in the present patient, the blood gas analysis revealed marked metabolic acidosis. Furthermore, this test can also be useful as means of assessing the severity of purging behaviors because acid–base disturbances reflect the direct physiological changes caused by these behaviors.

## CONCLUSION

In the present case, venous blood gas analysis was instrumental in detecting metabolic acidosis caused by excessive laxative use. Venous blood gas analysis may also be useful for detecting covert purging behaviors by revealing acid–base disturbances, even in cases where regular medication masks an electrolyte imbalance. This test may be integrated into the routine assessment of AN to assess the physiological changes resulting from purging behaviors. Future clinical studies are warranted to clarify the utility of venous blood gas analysis in the treatment of AN.

## AUTHOR CONTRIBUTION

*Conceptualization*: Takuto Ishida. *Resources*: Takuto Ishida. *Data curation*: Satoshi Miwa. *Writing of the first draft*: Satoshi Miwa. *Manuscript review and editing*: Takuto Ishida and Masafumi Mizuno. *Visualization*: Satoshi Miwa. *Supervision*: Takuto Ishida and Masafumi Mizuno. *Project administration*: Satoshi Miwa and Takuto Ishida. All the authors have read and agreed to submission of the current version of the manuscript.

## CONFLICT OF INTEREST STATEMENT

The authors declare no conflict of interest.

## ETHICS APPROVAL STATEMENT

N/A.

## PATIENT CONSENT STATEMENT

Written informed consent was obtained from the patient for the publication of this case report and the accompanying images.

## CLINICAL TRIAL REGISTRATION

N/A.

## Data Availability

The authors confirm that the data supporting the findings of this study are available within the article.
